# Higher Arc Nucleus-to-Cytoplasm Ratio during Sleep in the Superficial Layers of the Mouse Cortex

**DOI:** 10.3389/fncir.2017.00060

**Published:** 2017-08-23

**Authors:** Sakiko Honjoh, Luisa de Vivo, Hiroyuki Okuno, Haruhiko Bito, Giulio Tononi, Chiara Cirelli

**Affiliations:** ^1^Department of Psychiatry, University of Wisconsin-Madison Madison, WI, United States; ^2^Medical Innovation Center, Graduate School of Medicine, Kyoto University Kyoto, Japan; ^3^Department of Neurochemistry, Graduate School of Medicine, The University of Tokyo Tokyo, Japan

**Keywords:** AMPA receptors, sleep, synaptic depression, mouse, cortex

## Abstract

The activity-regulated cytoskeleton associated protein Arc is strongly and quickly upregulated by neuronal activity, synaptic potentiation and learning. Arc entry in the synapse is followed by the endocytosis of glutamatergic AMPA receptors (AMPARs), and its nuclear accumulation has been shown *in vitro* to result in a small decline in the transcription of the GluA1 subunit of AMPARs. Since these effects result in a decline in synaptic strength, we asked whether a change in Arc dynamics may temporally correlate with sleep-dependent GluA1 down-regulation. We measured the ratio of nuclear to cytoplasmic Arc expression (Arc Nuc/Cyto) in the cerebral cortex of EGFP-Arc transgenic mice that were awake most of the night and then perfused immediately before lights on (W mice), or were awake most of the night and then allowed to sleep (S mice) or sleep deprived (SD mice) for the first 2 h of the light phase. In primary motor cortex (M1), neurons with high levels of nuclear Arc (High Arc cells) were present in all mice, but in these cells Arc Nuc/Cyto was higher in S mice than in W mice and, importantly, ~15% higher in S mice than in SD mice collected at the same time of day, ruling out circadian effects. Greater Arc Nuc/Cyto with sleep was observed in the superficial layers of M1, but not in the deep layers. In High Arc cells, Arc Nuc/Cyto was also ~15%–30% higher in S mice than in W and SD mice in the superficial layers of primary somatosensory cortex (S1) and cingulate cortex area 1 (Cg1). In High Arc Cells of Cg1, Arc Nuc/Cyto and cytoplasmic levels of GluA1 immunoreactivities in the soma were also negatively correlated, independent of behavioral state. Thus, Arc moves to the nucleus during both sleep and wake, but its nuclear to cytoplasmic ratio increases with sleep in the superficial layers of several cortical areas. It remains to be determined whether the relative increase in nuclear Arc contributes significantly to the overall decline in the strength of excitatory synapses that occurs during sleep. Similarly, it remains to be determined whether the entry of Arc into specific synapses is gated by sleep.

## Introduction

The immediate early gene Arc (activity-regulated cytoskeletal protein, also known as Arg3.1) is strongly and quickly upregulated in excitatory glutamatergic neurons when synaptic activity increases, for instance during seizures, as well as after exploration of a novel environment or performance in many learning tasks (Link et al., 1995; Lyford et al., 1995; Guzowski et al., 1999, 2001; Vazdarjanova et al., 2006). Arc mRNA is transported into previously activated dendritic areas and most likely translated locally, and the spines of activated neurons contain Arc protein in the post-synaptic density (PSD) and in early endosomes (Steward et al., 1998; Steward and Worley, 2001; Moga et al., 2004; Rodríguez et al., 2005; Chowdhury et al., 2006). Arc is induced by high frequency electrical stimulation that leads to long-term potentiation (LTP), but not by low-frequency, long-term depression (LTD)-inducing stimulation (Steward et al., 1998). In frontal cortex, motor training increases Arc expression in a subset of excitatory neurons that later show persistent firing, and neither persistent firing nor the consolidation of motor learning occur without Arc (Ren et al., 2014). In many brain regions overtraining results in weaker Arc induction than new training (Kelly and Deadwyler, 2003), and in the hippocampus repeated presentation of the same stimulus leads to Arc reactivation in a progressively smaller neuronal population, despite no major changes in firing rates (Guzowski et al., 2006; Wang et al., 2006). Overall, these results indicate that Arc expression and neuronal activity are strongly coupled but the link is complex, because the levels of Arc also reflect neuronal plasticity and novelty.

Early studies indicated that Arc targets recently activated spines, suggesting a link with synaptic potentiation, but more recent experiments show that Arc leads to synaptic weakening. For instance, in CA1 pyramidal neurons Arc induction by a 5-min exposure to a novel environment does not affect synaptic function *per se*, but primes these cells for LTD. Specifically, after novelty exposure only Arc positive, but not Arc negative neurons undergo LTD in response to the *in vitro* activation of group 1 metabotropic glutamate receptors (mGluR-LTD), an effect that requires rapid protein synthesis (Jakkamsetti et al., 2013). Similarly, repeated exposure to the same environment leads to dendritic translation of Arc and synaptic weakening in Arc positive, but not Arc negative CA1 neurons (Jakkamsetti et al., 2013). Moreover, other studies found that Arc expression occludes LTD, and Arc translation is required for LTD mediated by mGluR-LTD (Rial Verde et al., 2006; Park et al., 2008; Smith-Hicks et al., 2010) and NMDA-dependent LTD (Plath et al., 2006, but see Park et al., 2008). By contrast, Arc is required for the consolidation, but not the induction, of LTP (Guzowski et al., 2000; Messaoudi et al., 2007). Early LTP is actually enhanced in Arc KO mice (Plath et al., 2006). The mechanism by which Arc may promote LTP stabilization remains unclear but may be indirect, via its primary role in promoting AMPA receptors (AMPARs) endocytosis and LTD (Shepherd and Bear, 2011).

Arc was shown to decrease synaptic strength by acting at the synapse and nuclear level. In the synapse, Arc interacts with endocytic proteins endophilin and dynamin and mediates the removal of labile surface AMPARs containing the subunits GluA1 and GluA2 (Chowdhury et al., 2006; Rial Verde et al., 2006). The exact mechanism remains unclear, since neither Arc nor endophilin and dynamin interact directly with AMPARs. However, it was recently shown that the same subdomain of Arc binds both CamKII and TARPγ2 (stargazin; Zhang et al., 2015), which is known to associate with AMPARs. After increased activity and LTP-inducing stimuli, Arc was shown to “inverse tag” the less activated spines, resulting in synapse-specific weakening (Okuno et al., 2012). In the nucleus, *in vitro* experiments found that Arc accumulation leads to small decreases (~20%) in the transcription of the GluA1 subunit of AMPARs, surface expression of GluA1-containing AMPARs, and amplitude of the miniature excitatory post-synaptic currents mediated by these receptors (Korb et al., 2013).

There is increasing evidence that sleep promotes a net decrease in synaptic efficacy to counteract the net synaptic potentiation that results from massive and ongoing wake-related learning, as demonstrated using molecular and electrophysiological measures of synaptic strength (Tononi and Cirelli, 2014). Moreover, a recent study employed serial block-face scanning electron microscopy to reconstruct ~7000 cortical spines in layer 2 of the mouse primary motor and sensory cortex. The goal was to test the prediction that sleep should lead to an overall shrinkage of most synapses, since morphological and functional measures of synaptic strength are strongly correlated. Indeed, a few hours of sleep led to an overall 18% decrease in the size of the axon-spine interface, the area of direct contact between axonal bouton and spine head (de Vivo et al., 2017). At the population level the sleep-related shrinkage of spines appeared to follow a scaling relation and was present in most (80%) but not all spines, sparing the largest ones and those lacking endosomes (de Vivo et al., 2017). Another recent study (Diering et al., 2017) measured the levels of GluA1- and GluA2-containing AMPARs in the PSD of forebrain synapses, and found an ~25% decrease after sleep as compared to after wake, consistent with previous findings in cortex and hippocampus (Vyazovskiy et al., 2008). This study also documented the crucial role of the immediate early gene *Homer1a* in sleep-dependent downscaling. *Homer1a*, like *Arc*, is upregulated during wake, but it accumulates inside the spines during sleep, when it activates constitutive mGluR5 signaling and promotes AMPARs endocytosis (Diering et al., 2017).

Here we asked whether Arc could also be involved in sleep-dependent synaptic weakening by testing whether sleep and wake affect its nuclear accumulation. In an early study we found that after 3–8 h of wake Arc was widely expressed throughout the cerebral cortex, hippocampus and striatum (Cirelli and Tononi, 2000). In the cerebral cortex, Arc expression was highest in layers 2/3 and 5/6, and appeared first in large pyramidal neurons of layer 5 and then in smaller neurons in other layers (Cirelli and Tononi, 2000). Moreover, Arc expression during wake depended on an intact noradrenergic system, since after lesions of the locus coeruleus awake animals showed low, sleep-like levels of Arc (Cirelli and Tononi, 2000). These early experiments linked Arc expression to wake and more specifically to the occurrence of plastic events promoted by the activation of the noradrenergic system. However, at that time the function of Arc in synaptic depression was unknown, and so was the specific role played in this process by Arc nuclear accumulation.

## Materials and Methods

### Animals

Adult male and female transgenic mice harboring mEGFP-Arc under the control of the *Arc*-promoter and 3′UTR were used (homozygous, P73–P112; Okuno et al., 2012). Investigators were blinded to sample condition. Sample size was based on past experience and pilot experiments. All animal procedures were used with approval from the National Institutes of Health Guide for the Care and Use of Laboratory Animals, and from Institutional Review Committees of the University of Tokyo Graduate School of Medicine. Facilities were reviewed and approved by the IACUC of the University of Wisconsin-Madison, and were inspected and accredited by the Association for Assessment and Accreditation of Laboratory Animal Care International (AAALAC).

### Experiments

**Figure 1:**
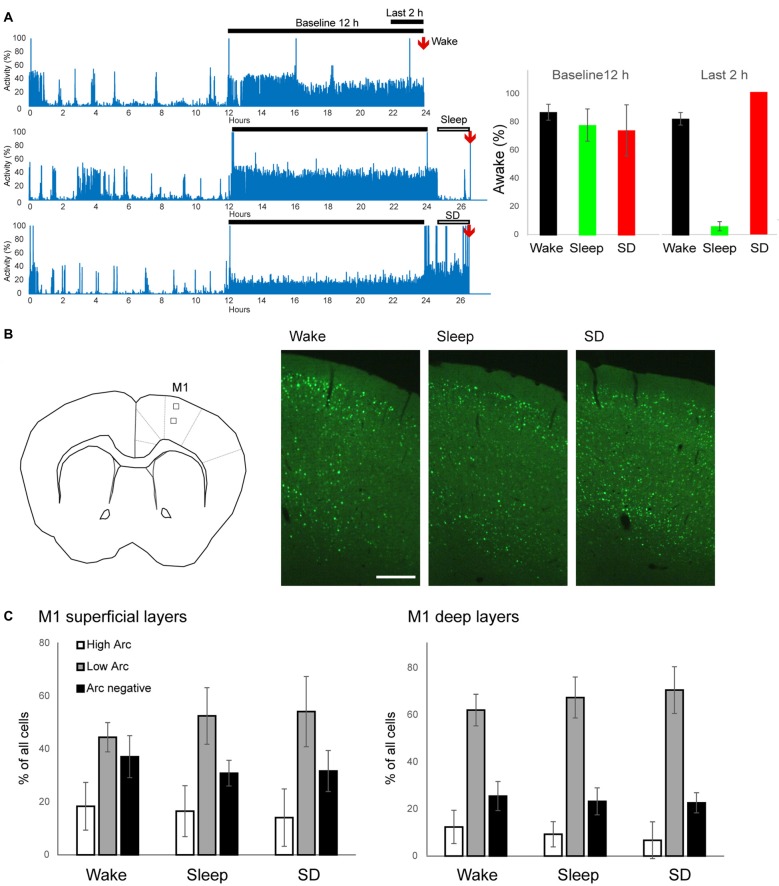
Experimental groups and EGFP-Arc expression in primary motor cortex (M1). **(A)** Left, representative motor activity recordings in one mouse for each experimental group. Right, quantification of time spent awake during the 12 h of baseline dark phase and during the last 2 h prior to perfusion (6 mice/group). **(B)** Left, schematic representation of imaging field in superficial and deep layers in M1 (left, square boxes). Right, examples of EGFP-Arc expression in frontal cortex in each experimental condition. Scale bar = 0.25 mm. **(C)** Quantification of EGFP-Arc expression levels in superficial and deep layers. The percentage of neurons with High Arc expression (white), Low Arc expression (gray) and without Arc expression (black) are shown. Bars and error bars show mean and standard deviation (6 mice/group).

Mice were singly housed in transparent Plexiglas cages (36.5 × 25 × 46 cm) for the duration of the experiment (light/dark 12:12, light on at 8 am, 23 ± 1°C; food and water available *ad libitum*). Motor activity was constantly monitored with an infra-red light equipped camera and quantified with 1-s resolution to assess sleep and wake behavior, as in previous studies (Maret et al., 2011; de Vivo et al., 2017). Mice were divided into three groups, wake (W), sleep (S) and sleep deprivation (SD). During the 12 h of the dark phase, all mice had free access to running wheels and novel objects were placed in the cage to promote exploration. W mice were perfused at the end of the dark phase, after spending most of the night awake (Figure 1A). The remaining two groups were followed for approximately two additional hours after lights on. In the S group, running wheels and novel objects were removed at the beginning of the light phase and mice were left undisturbed. S mice were selected based on quantification of motor activity and remotely inspected behavior, and perfused after having spent more than 90% of the last 2 h asleep (Figure 1A). In the SD group, running wheels and novel objects remained in the cage during the light period, to enforce wake. Mice were watched continuously to ensure that they did not fall asleep, and perfused after approximately 2 h after lights on. Thus, S and SD mice were perfused at the same circadian time, but after having spent most of the last 2 h in opposite behavioral states. In total four independent experiments were performed, for a total of six W mice, eight S mice and nine SD mice. A subset of these mice (6 mice/group) was used to measure the Arc nucleus/cytoplasm (Nuc/Cyto) ratio in superficial and in deep layers of primary motor cortex (M1) and another, partially overlapping set of mice (4 W, 6 S, 6 SD) was used to measure the Arc Nuc/Cyto ratio in neurons with high Arc expression in the superficial layers of primary sensory cortex (S1) and cingulate cortex (Cg1). In Cg1, the ratio between cytoplasmic GluA1 and Arc Nuc/Cyto was also measured (4 W, 6 S, 6 SD).

### Histology

Mice were transcardially perfused under deep anesthesia (3% isoflurane in oxygen) with 4% paraformaldehyde (PFA) with a 24-h post-fix in PFA. Brains were subsequently sectioned with a vibratome (Leica) into 30–40 μm thick coronal sections. Two or three sections (Bregma, +1.1 to +0.75 according to the Mouse Brain Atlas (Franklin and Paxinos, 2008)) were used for immunostaining. Sections were washed with PBS (pH 7.4) and treated with 0.3% Triton X-100 in PBS followed by a blocking solution (5% NGS, 1% BSA and 0.3% Triton X-100) for 1 h, incubated overnight in the blocking solution containing rabbit anti-GFP antibody (1:500, Thermo Fisher, A21311) at 4°C. For double-staining of GluA1 and EGFP-Arc, rabbit anti-Glutamate receptor 1 antibody (1:1000, Millipore, AB1504) and mouse anti-GFP antibody (1:500, Thermo Fisher, A-11120) were used. For BOBO-3 staining, sections were washed with PBS, incubated with 5% NGS PBS containing BOBO-3, a high-affinity nucleic acid stain (de Mazière et al., 1996; Harocopos et al., 1998; Lim et al., 2008; Tu et al., 2014; 1:1000, Thermo Fisher, B3586) for 2 h at room temperature, washed three times with PBS, mounted and air-dried. For double-staining of GluA1 and EGFP-Arc, sections were incubated with 5% NGS PBS containing secondary antibodies (1:500, Alexa-Fluor 488 Goat anti-mouse IgG, Thermo Fisher A11001 and Alexa-Fluor 568 Goat anti-rabbit IgG, Thermo Fisher, A11011) instead of BOBO-3. Low magnification images were acquired with Leica DMR/EC3 system (×5). To analyze the subcellular distribution of EGFP-Arc, 512 × 512 pixel single plane images (approximately 198 μm × 198 μm) were acquired with a confocal microscope (Olympus BX61W1, objective lens; PlanApo, ×60, NA 1.42; putative optical slice thickness FWHM, green = 0.48 μm, red = 0.55 μm) in both hemispheres.

### Image Analysis

Within the cortical area of interest, the specific image field was selected based on the cytoarchitecture as visualized by BOBO-3 staining. Under the protocol used here for fixation and immunohistochemistry, BOBO-3 stained strongly both the cytoplasm and the nucleoli but not the rest of the nucleus, consistent with higher affinity for RNA than for DNA. For the superficial layers II/III, the image field (198 μm × 198 μm) started from the bottom of layer I and spanned ventrally for ~200 μm. For the deep layers, the image field (198 μm × 198 μm) was centered around putative layer V, identified by the presence of very large pyramidal cells. Images for each image field were acquired with multiple laser power levels. To measure nuclear and cytoplasmic EGFP-Arc signal, regions of interest (ROIs) were drawn in the nucleus and the cytoplasm of each neuron based on BOBO-3 staining, avoiding nucleoli. Drawing was done manually by human observers blind to experimental conditions.

In M1, analysis was done in both superficial and deep layers and cells were classified into “High Arc”, “Low Arc” and “Arc negative” based on the EGFP-Arc signal in the nucleus. Based on previous studies, we assume that most, if not all, cells expressing Arc are neurons (Vazdarjanova et al., 2006). However, we cannot rule out that we included a few non-neuronal cells, since we did not perform immunohistochemistry to distinguish neurons from glia. First, we used a fixed set of acquisition parameters—laser power of 90 (a.u.), and PMT gain 720 (a.u.) to define as “High Arc” those cells whose nucleus was saturated under these conditions. The saturation of the Arc signal was determined for each single nucleus and saturation in one single pixel was sufficient to define the entire neuron as High Arc. Next, we used another set of acquisition parameters—laser power of 100 (a.u.), and PMT gain 720 (a.u.)—to divide the remaining, not saturated, cells into two groups, “Low Arc” cells and Arc negative (Arc-) cells, depending on whether the EGFP-Arc signal in their nucleus was higher or lower than the mean signal intensity of all pixels in the 198 × 198 μm image field. Note that the secondary distinction between Low Arc and Arc- cells (but not the primary distinction between High Arc cells and the rest of the cells) could be inconsistent across mice if their overall level of Arc staining in the image fields used for the analysis was highly variable. In M1, however, we found very similar overall levels of Arc expression across all mice, in both superficial and deep layers (data not shown). In Cg1 and S1, we only focused on High Arc neurons, whose identification was independent of overall levels of Arc staining. The Nuc/Cyto of the EGFP-Arc signal was calculated by using the mean signal intensity of nuclear ROI and cytoplasmic ROI in each cell. For each neuron, the EGFP-Arc signal was measured in the image acquired with the highest laser power just below the level that caused signal saturation. Based on BOBO-3 staining, cells showing clear nucleoli and Nuc/Cyto boundary were considered in focus. Cells out of focus or with a cytoplasm too thin to draw a reliable ROI based on BOBO-3 staining were not used.

In Cg1 and S1, only superficial layers were analyzed and the analysis focused on “High Arc” cells, identified using the criteria described above. In the images with GluA1 and EGFP-Arc double-staining, both signals, as well as cell body size and cell body shape, were considered to determine whether the cell was in focus. ROIs were drawn in the nucleus and the cytoplasm of a neuron to calculate the Arc Nuc/Cyto, as described above, and the GluA1 signal was obtained using the mean cytoplasmic ROI.

## Results

To study the effects of sleep and wake on Arc nuclear accumulation, we used a previously characterized EGFP-Arc transgenic line (Okuno et al., 2012). First, we confirmed in pilot experiments that in these mice, EGFP-Arc was widely expressed throughout the cerebral cortex, hippocampus and striatum after wake, and originally expressed EGFP-Arc was almost completely absent after 8 h of sleep (data not shown), consistent with prior results shown in rats (Cirelli and Tononi, 2000). Thus, we focused on the subcellular localization of EGFP-Arc after 2 h of sleep, before most of the protein had disappeared. We compared three groups of animals, wake (W), sleep (S) and sleep deprivation (SD) mice. All animals were well entrained to the 12 h light:12 h dark conditions, sleeping during most of the light phase (not shown) and staying awake at least 70% of the night (Figure 1A). In W mice, brain collection occurred just at the end of the dark phase, while S and SD mice were perfused at the same circadian time, approximately 2–2.5 h after lights on, after having spent most of the last 2 h asleep or awake, respectively (Figure 1A, right).

**Figure 2:**
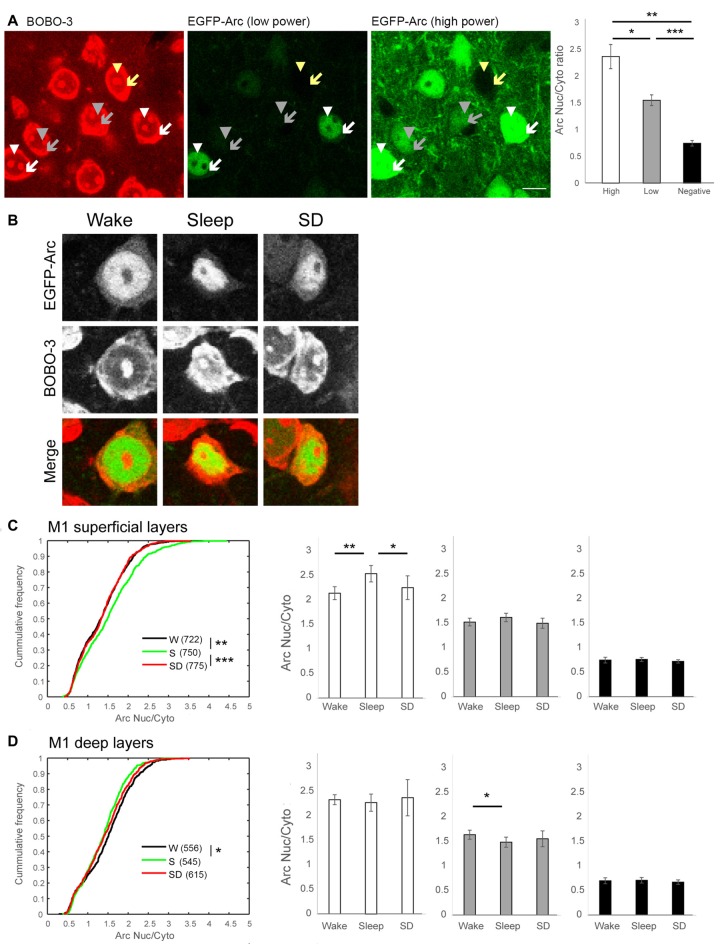
Subcellular pattern of EGFP-Arc expression in M1.** (A)** Representative images of BOBO-3 staining (left panel) and EGFP-Arc signal at two different laser power levels (middle and right panel) from the same field (S mouse). Arrowheads and arrows indicate nucleus and cytoplasm of neurons with High Arc expression (white), with Low Arc expression (gray) and without Arc expression (yellow), respectively. Scale bar = 10 μm. A positive correlation between Arc nucleus/cytoplasm (Nuc/Cyto) ratio and nuclear Arc signal was observed in all mice. Right, bars and error bars show mean and standard deviation of mean Arc Nuc/Cyto in High Arc, Low Arc and Arc negative neurons in each mouse. *p = 4.86e-11, **p = 2.23e-15, ***p = 3.20e-18, paired *t*-test using mean values for each mouse. **(B)** Representative images of subcellular localization of EGFP-Arc in each experimental group. EGFP-Arc images were acquired with the same parameters in all three conditions. **(C,D)** Summary of changes in Arc Nuc/Cyto in superficial and in deep layers. Left, cumulative frequency plots of Arc Nuc/Cyto ratio of all neurons analyzed from six mice per group (number of cells in parenthesis) *p < 0.001, **p < 0.0005, ***p < 0.000005, two-sample Kolmogorov-Smirnov test using Arc Nuc/Cyto ratio of all analyzed neurons. Right, higher Arc Nuc/Cyto after 2 h of sleep is observed in neurons with High Arc expression. Mean and standard deviation (6 mice/group). *p < 0.05, **p < 0.01, unpaired *t*-test using mean value of each mouse.

We focused first on M1 (Figure 1B), where we recently found ultrastructural evidence for sleep-dependent synaptic weakening (de Vivo et al., 2017). Most (~70%) M1 neurons showed some level of EGFP-Arc expression, independent of experimental group (Figure 1B, right). We classified neurons as “High Arc”, “Low Arc” and “Arc negative” based on their nuclear signal (see "Materials and Methods" Section for details) and found that the percentage of cells in each category did not differ across groups, neither in the superficial (II/III) nor in the deep (V) layers (Figure 1C). Similarly, in all groups expression levels of EGFP-Arc were highly heterogeneous across cells, but showed the same general positive correlation between Arc nuclear levels and Arc Nuc/Cyto; that is, Arc Nuc/Cyto was greater than 2 in High Arc neurons and smaller than 1.5 in Low Arc neurons (Figure 2A). Furthermore, Arc Nuc/Cyto was higher in S mice compared to W mice and, most crucially, higher in S mice than in SD mice collected at the same circadian time. This difference was found in superficial but not in deep layers of M1 and was specific for High Arc neurons. In these cells, which presumably had been activated more strongly due to larger increases in neuronal activity and/or plasticity, Arc Nuc/Cyto was ~15% higher after sleep than after SD (W 2.14, S 2.54, SD 2.25; Figures 2B,C). In deep layers, Arc Nuc/Cyto was slightly (5%) smaller in S mice relative to W mice, but not relative to SD mice collected at the same circadian time, and the difference was only seen in Low Arc neurons, making this result difficult to interpret (Figure 2D).

**Figure 3:**
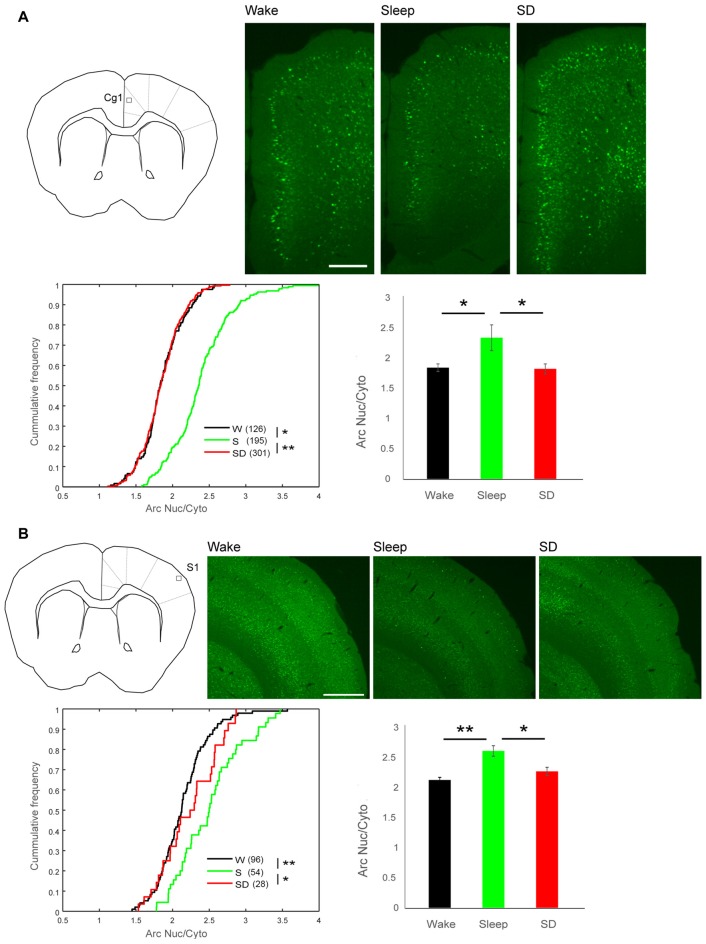
Arc Nuc/Cyto in superficial layers of cingulate cortex area 1 (Cg1) and primary somatosensory cortex (S1).** (A)** Top left, schematic representation of imaging field in superficial layers of Cg1. Top right, examples of EGFP-Arc expression in each group. Scale bar = 0.25 mm. Bottom left, cumulative frequency plots of Arc Nuc/Cyto in High Arc neurons in superficial layers of Cg1 (number of cells in parenthesis) *p = 2.47–22, **p = 9.23e-37, two-sample Kolmogorov-Smirnov test using Arc Nuc/Cyto ratio of all analyzed neurons. Right, mean and standard deviation (*W* = 4 mice, *S* = 6, SD = 6). *p < 0.01, unpaired *t*-test using mean value of each mouse. **(B)** Top left, schematic representation of imaging field in superficial layers of S1. Top right, examples of EGFP-Arc expression in each group in S1. Scale bar = 0.5 mm. Bottom left, cumulative frequency plots of Arc Nuc/Cyto in High Arc neurons in superficial layers of S1 (number of cells in parenthesis). *p = 0.0087, **p = 4.00e-07, unpaired *t*-test using Arc Nuc/Cyto ratio of all analyzed neurons. Right, mean and standard deviation (*W* = 4 mice, *S* = 6, SD = 6). *p < 0.05, **p < 0.01, unpaired *t*-test using mean value of each mouse.

Next, we determined whether High Arc neurons in the superficial layers of other cortical areas also showed higher Arc Nuc/Cyto during sleep than during wake. We focused on the primary somatosensory area (S1), where we also found ultrastructural evidence for sleep-dependent synaptic scaling (de Vivo et al., 2017), as well as on the higher order Cg1 (Figures 3A,B). In S1, the number of High Arc neurons in superficial layers was lower than in Cg1 and M1 (Cg1 vs. S1, *p* < 1*10E-4; M1 vs. S1, *p* < 5*10E-5; paired *t*-test, 16 mice). Moreover, in Cg1 and S1 the number of High Arc neurons in superficial layers tended to be lower in sleep than in either spontaneous wake or SD, suggesting that contrary to M1, in these regions the sleep-associated decline in Arc expression may occur already after 2 h of sleep (mean N of cells in Cg1, W: 126 (*n* = 4), S: 195 (*n* = 6), SD: 301 (*n* = 6); W vs. S *p* = 0.6449; W vs. SD *p* = 0.1794; S vs. SD *p* = 0.0777; mean N of cells in S1, W: 96 (*n* = 4), S: 45 (*n* = 6), SD: 28 (*n* = 6); W vs. S *p* = 0.0263; W vs. SD *p* = 0.0076; S vs. SD *p* = 0.4149, unpaired *t*-test). Despite these differences in absolute Arc expression, both areas showed higher Arc Nuc/Cyto after sleep in High Arc neurons. Specifically, relative to SD, the increase was ~28% in Cg1 (W 1.85, SD 1.83, S 2.35; sleep vs. wake *p* < 0.01, sleep vs. SD *p* < 0.001, unpaired *t*-test) and 15% in S1 (W 2.12, SD 2.27, S 2.61; sleep vs. wake *p* < 0.01, sleep vs. SD *p* < 0.05, unpaired *t*-test). As described in the Methods, the identification of High Arc cells is not affected by the overall levels of Arc staining.

**Figure 4:**
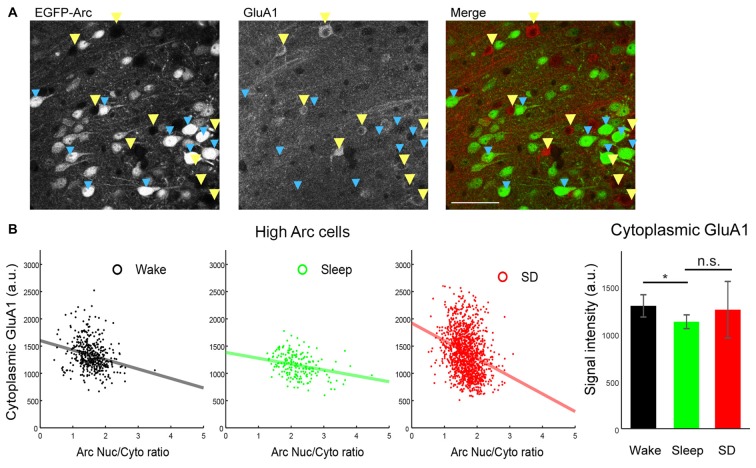
Negative correlation between Arc Nuc/Cyto and cytoplasmic GluA1 levels in High Arc neurons of Cg1 superficial layers. ** (A)** Representative images of double-staining of EGFP-Arc and GluA1 in Cg1 (SD mouse). Blue arrowheads: High Arc neurons, yellow arrowheads: Arc negative neurons. **(B)** Left, negative correlation between Arc Nuc/Cyto and cytoplasmic GluA1 signal. All neurons analyzed are shown. Right, mean and standard deviation of cytoplasmic GluA1 levels. *p < 0.05, unpaired *t*-test using mean value of each mouse.

Finally, we performed double staining of EGFP-Arc and GluA1 and measured Arc Nuc/Cyto and cytoplasmic GluA1 levels in High Arc neurons of Cg1 (Figure 4A). In all experimental groups we found a negative correlation between Arc Nuc/Cyto and cytoplasmic GluA1 (S, *R* = −0.2899, *p* < 5*E-5, *n* = 205 cells; W, *R* = −0.2427, *p* < 1*E-04, *n* = 418; SD, *R* = −0.2809, *p* < 1*E-24, *n* = 1289; Figure 4B), consistent with previous evidence (Korb et al., 2013). Moreover, cytoplasmic GluA1 levels in High Arc cells were lower after sleep than after spontaneous wake, but did not differ significantly between sleep and SD, due to high variability in the latter group (Figure 4B, right).

## Discussion

In this study we found that Arc moves to the nucleus during both sleep and wake, but its nuclear to cytoplasmic ratio increases with sleep in the superficial layers of several cortical areas. This change in Arc dynamics could be to several factors that we cannot tease apart, since we used a relative measure, the Arc Nuc/Cyto, taken at one single time point (a few hours of sleep or wake). Thus, the relative increase in nuclear Arc with sleep is likely to reflect a combination of multiple factors, nuclear accumulation, cytoplasmic and nuclear degradation, as well as dendritic translocation, all of which can be at least partially activity-dependent and may follow different time courses. Arc nuclear content was previously reported to be bi-phasically regulated by neuronal activity: a 30-min exposure to BDNF or bicuculline *decreases* Arc nuclear localization, while 8 h of BDNF or bicuculline lead to the active import of Arc into the nucleus, followed by cell-wide synaptic weakening (Korb et al., 2013). Furthermore, during exposure to a novel environment Arc shows a time-dependent increase in nuclear localization in both hippocampus and somatosensory cortex, appearing in the cytoplasm in the first 30 min, and then gradually moving to the nucleus in the next 2–8 h (Korb et al., 2013). Thus, one possibility was that once *Arc* is strongly induced, Arc nuclear accumulation occurs with the passage of time, independent of sleep and wake. Alternatively, Arc levels may be subject to rapid degradation as reported previously (Greer et al., 2010; Mabb et al., 2014), and this may somehow bias residual amounts of Arc in the nucleus vs. the cytoplasm. Our results are consistent with both ideas, as a large number of neurons with strong nuclear Arc expression were found in all experimental groups. W mice spent most of the previous 12 h awake, and SD mice were awake for two more hours, indicating that Arc entry in the nucleus occurs during prolonged wake. Yet, our results also suggest that as compared to SD, in the superficial layers of three different cortical areas—M1, S1, Cg1—sleep promotes the increase of Arc levels in the nucleus relative to the cytoplasm. Since the brains of S and SD mice were collected at the same circadian time, this differential effect cannot be ascribed to a circadian mechanism.

A role in sleep-dependent synaptic weakening was recently shown for another immediate early gene, *Homer1a* (Diering et al., 2017). The induction of both *Homer1a* and *Arc* is coupled with neural activity and occurs during wake, not during sleep. High noradrenaline levels and low adenosine levels, which are typical of spontaneous wake, prevent Homer1a from entering the spines and from triggering AMPARs endocytosis, at least when mice are awake for just a few hours (Diering et al., 2017). Whether this is also the case for Arc is unknown, and will be tested in future experiments. We know, however, that in CA1 pyramidal neurons Arc induction by a 5-min exposure to a novel environment does not affect synaptic function *per se*, but primes these cells for subsequent LTD. Specifically, after novelty exposure only Arc positive neurons, but not Arc negative, undergo LTD in response to the *in vitro* activation of group 1 mGluR, an effect that requires rapid protein synthesis (Jakkamsetti et al., 2013). Similarly, repeated exposure to the same environment leads to dendritic translation of Arc and synaptic weakening in Arc positive, but not Arc negative CA1 neurons (Jakkamsetti et al., 2013). Thus, it is possible that Arc induction during wake tags specific spines and/or neurons for future synaptic depression when conditions conducive to this process, such as sleep, occur. Furthermore, Arc induction during wake depends on an intact noradrenergic system, and its nuclear and/or synaptic accumulation may also be sensitive to noradrenaline levels, perhaps in the same way Homer1a entry into the synapse is gated by noradrenaline.

Greater relative nuclear accumulation of Arc during sleep was seen only in superficial layers, although number of High Arc cells and Nuc/Cyto ratio were comparable in layers 2/3 and 5/6. A previous study assessed the early phase of Arc activation by measuring mRNA levels after rats performed the same trained behavior in two different rooms, but did not measure the subsequent accumulation of Arc protein in nucleus and cytoplasm. It found largely non-overlapping populations of Arc positive neurons in the CA1 region of the hippocampus, an expected finding given the ability of these cells to reflect spatial context (Takehara-Nishiuchi et al., 2013). However, equally distinct patterns of *Arc* induction were found in the superficial, but not in the deep layers of posterior parietal and granular insular cortex (Takehara-Nishiuchi et al., 2013). *Arc* induction remained variable in layers 2/3 after hippocampal lesions, and it was reduced but not abolished when rats presumably had the same exact experience twice, that is, when they performed the same task twice in the same room. The authors suggested that the variable patterns of *Arc* induction may reflect the fact that network activity in superficial layers is more unstable and likely to “drift” over time, perhaps because it depends less on afferent stimuli and more on local multiple connections. In general, superficial layers are assumed to be more “plastic” (Diamond et al., 1994; Fox, 2002; Jiang et al., 2007), and thus one could speculate that neurons in these layers are also more prone to sleep-dependent synaptic changes.

We found that the levels of GluA1 in the soma tend to decrease during sleep relative to wake, a finding in keeping with previous reports where active GluA1 synthesis was linked to higher activity (Ju et al., 2004). We also found that, independent of behavioral state, High Arc cells in cingulate cortex showed an apparent negative correlation between relative nuclear accumulation of Arc and cytoplasmic levels of GluA1. In the same area, we also noticed that Arc negative cells consistently showed higher cytoplasmic GluA1 levels than High Arc cells, across all behavioral states (data not shown). These observations are consistent with previous evidence suggesting that Arc expression negatively correlated with glutamate receptor levels in general (Shepherd and Bear, 2011; Okuno et al., 2012; Korb et al., 2013), but further studies are needed to obtain direct mechanistic insights for this relationship. In theory, cytoplasmic levels of AMPARs could also affect Arc expression, since *in vitro* the pharmacological block of AMPARs potentiates activity-dependent *Arc* transcription (Rao et al., 2006). However, the pharmacological block of NMDA receptors has the opposite effect on Arc expression, and when neurons are stimulated with the endogenous neurotransmitter glutamate, mimicking more closely what happens *in vivo*, the effects on Arc levels are small, and positive (Rao et al., 2006).

In summary, we found that Arc moves to the nucleus after long periods of spontaneous nocturnal wake, as well as when long spontaneous wake is followed by a few hours of sleep or SD. We also found that 2 h of sleep leads to an increase in Arc nuclear to cytoplasmic ratio as compared to 2 h of SD. This effect is small (~15%–30% increase with sleep) and restricted to superficial layers of the cortex and its functional significance, perhaps to inhibit the transcription of GluA1, remains to be established.

## Author Contributions

SH and LdV collected and analyzed data; HO and HB provided experimental tools; SH, LdV, GT and CC designed the experiments; SH, HB, GT and CC wrote the article.

## Conflict of Interest Statement

GT consults for Philips Respironics and is involved in a research study in humans supported by Philips Respironics. This study is not related to the work presented in the current manuscript. The other authors declare that the research was conducted in the absence of any commercial or financial relationships that could be construed as a potential conflict of interest.
